# Provision of safe patient care during the COVID-19 pandemic despite shared patient rooms in a tertiary hospital

**DOI:** 10.1186/s13756-022-01091-1

**Published:** 2022-04-21

**Authors:** Astrid Füszl, Lukas Bouvier-Azula, Miriam Van den Nest, Julia Ebner, Robert Strassl, Cornelia Gabler, Magda Diab-Elschahawi, Elisabeth Presterl

**Affiliations:** 1 Department of Infection Control and Hospital Epidemiology, Medical University of Vienna, Vienna, Austria; 2 Department of Laboratory Medicine (Division of Clinical Virology), Medical University of Vienna, Vienna, Austria; 3 Research, Documentation and Analysis (RDA), Medical University of Vienna, Vienna, Austria

**Keywords:** Testing strategy, Universal screening, COVID-19, Healthcare-associated COVID-19, Nosocomial outbreak, Infection prevention and control

## Abstract

**Background:**

The COVID-19 pandemic has resulted in the disruption of healthcare systems. Vienna General Hospital (VGH), a tertiary hospital located in Austria, ran at almost full capacity despite high levels of community SARS-CoV-2 transmission and limited isolation room capacity. To ensure safe patient care, a bundle of infection prevention and control (IPC) measures including universal pre-admission screening and serial SARS-CoV-2 testing during hospitalization was implemented. We evaluated whether testing as part of our IPC approach was effective in preventing hospital outbreaks during different stages of the pandemic.

**Methods:**

In this retrospective single center study, we analyzed the SARS-CoV-2 PCR test results of cases admitted to VGH between a low (15/05/2020–01/08/2020) and a high incidence period (15/09/2020–18/05/2021). Outcomes were the diagnostic yield of (a) admission screening, (b) the yield of serial testing during hospitalization and (c) the occurrence of healthcare-associated COVID-19 (HA-COVID-19) and SARS-CoV-2 related hospital outbreaks.

**Results:**

The admission test positivity rate was 0.2% during the low and 2.3% during the high incidence phase. Regarding test conversions, 0.04% (low incidence phase) and 0.5% (high incidence phase) of initially negative cases converted to a positive test result within 7 days after admission The HA-COVID-19 incidence rate per 100,000 patient days was 1.0 (low incidence phase) and 10.7 (high incidence phase). One COVID-19 outbreak affecting eight patients in total could be potentially ascribed to the non-compliance with our IPC protocol.

**Conclusion:**

Testing in conjunction with other IPC measures enabled the safe provision of patient care at a hospital with predominantly shared patient rooms despite high case numbers in the community.

**Supplementary Information:**

The online version contains supplementary material available at 10.1186/s13756-022-01091-1.

## Introduction

As of September 2021, coronavirus disease 2019 (COVID-19) has affected over 229 million people and cost over 4.7 million people their lives [1]. Although the way of reporting COVID-19 deaths differs by location, estimates suggest that the total number of deaths attributable indirectly to COVID-19 is considerable. One reason for this seems to be the disruption of healthcare systems, with many health care centers closing down or only providing critical care due to a high prevalence of healthcare related transmissions and reluctance to seek care among patients.

Hospitals are high-risk settings for severe acute respiratory syndrome coronavirus 2 (SARS-CoV-2) transmission. Not only can the virus spread rapidly in the absence of adequate infection prevention and control (IPC) measures [2, 3], but patients are also at higher risk of developing COVID-19 related complications due to their underlying conditions. Hospitals are not always designed in a way to minimize the risk of patient-to-patient transmission (e.g. having limited isolation capacities) [4]. To keep medical operations running and ensure safe patient care, various IPC strategies have been developed tailored to the COVID-19 population prevalence, changes in transmissibility due to the emergence of new variants, the speed of spread, testing capacity, the availability of personal protective equipment, hospital infrastructure and vaccination coverage rates [5]. They usually include physical distancing and universal masking regulations, contact, droplet and/or airborne precautions during COVID-19 patient care, isolation of suspected/confirmed cases and COVID-19 vaccination of healthcare workers [6].

Another pillar of prevention concepts is the early detection of COVID-19 cases through testing. Hospital testing strategies vary. While some facilities rely on testing of symptomatic patients only, others combine diagnostic testing with screening because SARS-CoV-2 can also be transmitted by asymptomatic and pre-symptomatic individuals [7–10]. Depending on the setting, patients are either universally screened on admission [11], or screening is applied to specific patient groups, e.g. immunocompromised patients [12], patients who undergo elective surgery [13, 14] or pregnant women admitted for delivery [15, 16]. In addition to pre-admission screening, some facilities also re-test patients during hospitalization [17–19]. Repeated testing can help identify cases that are initially missed during their incubation period, cases with a false negative initial test result and cases who acquire the infection at the hospital.

At the beginning of the pandemic, our institution was a “non-COVID-19” hospital and all COVID-19 cases were transferred to other facilities. However, quickly rising case numbers in the community soon necessitated a change of this strategy. To provide care to COVID-19 patients, designated COVID-19 wards with a focus on intensive care treatment including extracorporeal life support were established. To ensure safe patient care against the backdrop of a high COVID-19 population prevalence and only few single-occupancy patient rooms, universal pre-admission screening in addition to re-testing during hospitalization were implemented. Being a tertiary care hospital, it was vital to maintain medical care for patients that required specialized treatment.

Although pre-admission SARS-CoV-2 screening and repeated testing have been advocated [20, 21], little is known about the diagnostic yield, the appropriate time interval between tests [17, 18, 22] and the potential for preventing hospital outbreaks. The aim of our study was to quantify the number of patients with a positive admission test and to evaluate whether serial testing during hospitalization yielded additional cases in the context of varying levels of community transmission. We also analyzed whether our testing strategy—used in conjunction with other IPC measures—was effective in preventing HA-COVID-19 and hospital outbreaks in a setting with limited isolation capacities.

## Methods

### Study design

In this retrospective single-center observational study, we analyzed (a) the positivity rate among patients tested prior to admission, (b) the conversion rate among serially tested inpatients (including information regarding the source of infection: community versus hospital) and (c) the occurrence of nosocomial COVID-19 related outbreaks. These analyses were conducted separately for a low and a high incidence phase.

### Setting

#### Community setting

On February 25th 2020, the first two cases of COVID-19 were confirmed in Austria. Case numbers quickly increased thereafter, resulting in the first lockdown on March 16th. After an easing of restrictions from April 2020 onwards and steadily low numbers during summer, there was a renewed rapid increase in the number of infections commencing in early fall. This rise was declared a second wave on September 13th, which resulted in another full national lockdown on November 17th 2020 that was extended until February 2021. Contact restrictions were eased thereafter. Alongside, strict community-based prevention measures were established, including the use of N95 respirators in many public areas and the mandatory provision of a negative SARS-CoV-2 test result, proof of vaccination or disease recovery for the use of certain services. Case numbers temporarily dropped, but generally remained high until the beginning of summer.

#### Hospital setting

Vienna General Hospital (VGH) is a 1728 bed tertiary hospital with a single bed capacity of 3% located in Vienna, Austria. During the first Austrian wave and government-initiated lockdown, elective procedures and non-urgent outpatient visits were postponed, resulting in fewer admissions. In April 2020, the hospital gradually returned to routine medical operations.

An area for initial assessment was set up at entry points, separating symptomatic from asymptomatic patients. Additional IPC measures included access restrictions for visitors, universal masking requirements for healthcare workers (HCWs) and visitors (medical masks at the beginning, later upgraded to N95 respirators), the use of personal protective equipment (PPE) during COVID-19 patient care (N95 respirators for general patient care, N99 respirators for aerosol-generating procedures, gown, gloves and googles/face shield), the creation of designated COVID-19 wards, isolation of suspected or confirmed cases, contact tracing and intensified environmental cleaning and disinfection. From January 2021 onwards, personnel at VGH was vaccinated.

The particular challenges related to routine medical operations in a pandemic setting included infrastructural deficits (the vast majority of patient rooms are three-bed rooms) and PPE shortages (particularly N95 respirators at the early stages of the pandemic). However, for the most part, triple rooms were only occupied by two patients during 2020.

#### Testing strategy at Vienna General Hospital

At the beginning of the pandemic, only symptomatic suspected COVID-19 cases were tested, using reverse transcription polymerase chain reaction (RT-PCR) technique. To counter the increased risk of nosocomial SARS-CoV-2 transmission in the context of resumed full operations, multi-bed rooms and PPE supply chain issues, universal pre-admission screening and re-testing of all hospitalized patients were added to the testing strategy. A negative pre-admission test (max. 72 h old) as well as compliance with pre-admission home quarantine between sample collection and admission were prerequisites for elective admissions. Additionally, the testing strategy foresaw re-testing of continuously asymptomatic patients 3 days after admission. Per testing protocol, early-interval re-testing should preferably be conducted using PCR, but antigen tests were also accepted. Patients were also re-tested prior to invasive, aerosol-generating procedures (e.g. intubation, bronchoscopy) and when they developed symptoms compatible with COVID-19. Further, the IPC protocol recommended weekly SARS-CoV-2 testing of inpatients with a long-term hospital stay. Patients admitted for acute conditions were isolated and managed as possible COVID-19 cases until a negative test result was confirmed. Alongside, healthcare staff was universally screened for SARS-CoV-2 on a weekly basis. These measures were maintained throughout the whole study period, irrespective of SARS-CoV-2 vaccination rates among HCWs and patients.

Nasopharyngeal swabs were performed by specially trained HCWs throughout the two study periods. Tests were analyzed by the hospital’s clinical virology laboratory. Depending on the assigned patient priority level and need for a quick test result, different RT-PCR test systems were used for the detection of SARS-CoV-2 RNA (emergency/vital indication: Cepheid GeneXpert; urgent: Qiagen NeuMoDx N96 and N288; routine care: Roche Cobas 6800, Abbott AlinityM as well as manual PCR based on Charité protocol [23]. A guideline issued by the Austrian Ministry of Health regarding the release of confirmed COVID-19 cases from home isolation listed a cycle threshold (Ct) cut-off above 30 as one of the criteria [24]. Following this, repeated Ct-values above 30 were used to define past infection in our study population.

### Variables

#### Definition of low and high incidence

Low incidence was defined as a 7-day incidence of less than 50 per 100,000 population (defined as a critical infection rate threshold by the German government) [25]. Everything above this threshold was categorized as high incidence. Incidence rates between May 2020 and May 2021 per 100,000 population were obtained from the Austrian Agency for Health and Food Safety (AGES), using their publicly available COVID-19 dashboard [26]. Vienna was chosen as the point of reference to reflect the main catchment area of patients receiving care at VGH.

For the comparison of low and high incidence, the following two time periods were chosen:

- 15/05/2020–01/08/2020 (7-day incidence ranging from 6 to 18 per 100,000 population)
- 15/09/2020–18/05/2021 (7-day incidence ranging from 53 to 443 per 100,000 population)

The corresponding epidemic curve showing laboratory confirmed COVID-19 cases in Vienna is presented in Additional file 1: Fig. S1 (Supplement).

#### Definitions of community-associated (CA) COVID-19, healthcare-associated (HA) COVID-19 and nosocomial outbreak of COVID-19

Table 1 shows a summary of COVID-19 surveillance definitions (for the identification of the source of infection) and outbreak criteria, using information provided by the European Centre for Disease Prevention and Control (ECDC) and Public Health England [27, 28].

**Table 1:** Definition of COVID-19 according to the source of infection and nosocomial outbreak criteria

Community-associated COVID-19 (CA-COVID-19)	(a) Symptoms present on admission or with onset on day 1 or 2 after admission
	(b) Symptom onset on day 3–7 and a strong suspicion of community transmission
Indeterminate association (IA-COVID-19)	Symptom onset on day 3–7 after admission, with insufficient information regarding the source of infection to assign to another category
Probable healthcare-associated COVID-19 (HA-COVID-19)	(a) Symptom onset on day 8–14 after admission
	(b) Symptom onset on day 3–7 and a strong suspicion of healthcare transmission
Definite healthcare-associated COVID-19 (HA-COVID-19)	Symptom onset on day > 14 after admission
SARS-CoV-2-related hospital outbreak	Two or more test-confirmed COVID-19 cases among hospitalized patients with (a) illness onset after at least 8 days after hospital admission (for at least one of the cases) and (b) establishment of an epidemiological link between the cases (exposure event)

#### Analysis of the admission test positivity rate

To analyze how many patients had a positive admission screening test, hospitalized patients with at least one SARS-CoV-2 PCR test result were included. Tests conducted within 3 days prior to up until 1 day after admission were considered admission tests.

#### Analysis of test conversions after admission

To analyze how many additional likely CA-COVID-19 cases occurred during hospitalization, test results of inpatients with at least two SARS-CoV-2 PCR test results (negative admission test, second test within 7 days) were analyzed. To identify potential HA-COVID-19 cases, hospitalized patients with any positive SARS-CoV-2 PCR test result obtained on day eight or later were analyzed. Patients who had previously—at any given time—tested positive at VGH were excluded from the analysis to avoid distorting the results with non-infectious post-COVID-19 patients who can intermittently test positive for prolonged periods [29, 30]. Patient records were then reviewed to obtain additional clinical information. Patients with a newly acquired infection were classified according to COVID-19 surveillance  definitions by ECDC.

### Statistical methods

Descriptive statistics—for continuous variables means and standard deviations or medians und interquartile ranges (IQR) as appropriate, for categorical variables counts and percentages—were calculated using R Studio software (R Core Team, version 4.0.2). For HA-COVID-19 cases, incidence rates per 100,000 patient days were calculated as well.

## Results

We compiled a table that summarizes the most important findings (e.g. admission test positivity rate, yield of serial testing during hospitalization, HA-COVID-19 and SARS-CoV-2 outbreaks at our facility), comparing the low and high incidence phase (Table 2).

**Table 2:** Comparison of testing patterns and test results between the low and high incidence period

	Low incidence period	High incidence period
Patients with admission screening	98.0% (13,011/13,284)	93.8% (41,142/43,850)
Admission screening positivity rate	0.2% (22/13,284)	2.3% (945/41,142)
Conversion rate within 7 days after admission	0.04% (2/4,734)	0.5% (75/15,928)
Patients retested within 7 days after admission	36.4% (4,734/12,989)	39.6% (15,928/40,197)
Median length of stay (retested)	5.2 days (IQR 3.2–9.6)	6.1 days (IQR 3.6–11.0)
Median length of stay (not retested)	2.1 days (IQR 1.1–4.3)	2.1 days (IQR 1.1–3.6)
HA-COVID-19 incidence rate per 100,000 patient days	1.0	10.7
Number of CA-COVID-19 cases	1	11
Number of cases with an indeterminate association	1	31
Number of probable HA-COVID-19 cases	0	13
Number of definite HA-COVID-19 cases	1	23
Number of SARS-CoV-2 hospital outbreaks	0	5*

^*^Of these five outbreaks, four only involved two patients that shared a room at the hospital

### Low incidence phase

#### Characteristics of patients testing positive within 7 days after admission

Among repeatedly tested hospitalized patients with a negative admission test, two (0.04%) out of 4734 converted to a positive test result within 7 days after admission.

One of the two SARS-CoV-2 positive patients was admitted for elective surgery and had an already known COVID-19 infection that had been diagnosed elsewhere (initial sample not analyzed at VGH). For the second inpatient who subsequently tested positive, no further information could be retrieved.

#### Classification of patients (CA-COVID-19 versus HA-COVID-19) testing positive at any time during hospitalization

One patient was classified as CA-COVID-19, one as IA-COVID-19 and one as definite HA-COVID-19. For the HA-COVID-19 case, frequent visits by relatives were identified as a potential source of infection.

#### Outbreak characteristics

No nosocomial COVID-19 outbreak occurred during this period.

### High incidence phase

#### Characteristics of patients testing positive within 7 days after admission

Among repeatedly tested hospitalized patients with a negative admission test, 75 (0.5%) out of 15,928 converted to a positive test result within 7 days after admission.

**Fig. 1:**
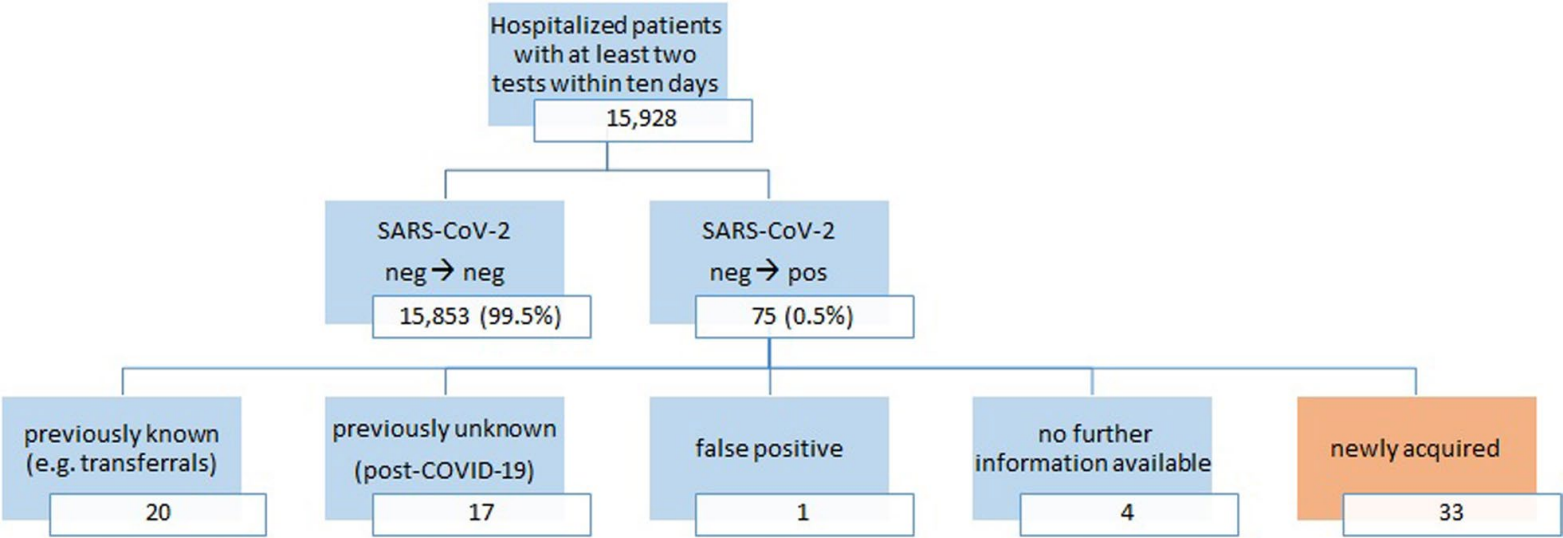
Patient record review of hospitalized patients testing positive within 7 days after admission (high incidence period). Displayed percentages refer to the total number of 15,928 hospitalized patients who were tested at least twice (starting with a negative pre-admission test), using RT-PCR

A detailed breakdown of patients testing positive within 7 days is presented in Fig. 1. Altogether 20 (26.7%) out of these 75 cases were already known COVID-19 cases (e.g. COVID-19 patients who were transferred from another hospital, with a negative test on arrival at the VGH, but a subsequent positive test at our facility). This was probably due to the fact that COVID-19 patients can remain positive, intermittently, for extended periods [29, 30]*.* Next, 17 (22.7%) patients were previously unknown (no further information regarding COVID-19 found in their medical records), but likely post-COVID-19 cases as they presented with consistently high Ct-values over 30 in follow-up testing. One case was described as having a false positive test result. For four patients, no further clinical information could be retrieved. The remaining 33 (44.0%) cases had a newly acquired infection, which represented 0.2% (33/15,928) of all hospitalized patients re-tested within 7 days. Altogether six cases were symptomatic at the time of diagnosis.

#### Classification of patients (CA-COVID-19 vs. HA-COVID-19) testing positive at any time during hospitalization

Overall, 11 cases were classified as CA-COVID-19 and 31 as IA-COVID-19. This was due to the lack of further information regarding the source of infection. Among the 13 cases classified as probable HA-COVID-19, one tested positive within 3–7 days after admission. He was part of a suspected hospital outbreak with an epidemiological link between other SARS-CoV-2 positive inpatients. Two patients tested positive 6 days after hospital discharge, but shared the same room at the hospital. The remaining 10 tested positive 8–14 days after admission. The other 23 cases with definite HA-COVID-19 tested positive > 14 days after admission. The source of infection could not be identified in most instances. However, among some of these definite HA-COVID-19 cases, a patient occupying the same room or a visitor had previously tested positive. In one case, the patient may have acquired the infection from a HCW previously testing positive.

#### Outbreak characteristics

Following our outbreak criteria, five hospital outbreaks were identified. Four of these outbreaks were confined to one room occupied by two patients. In the fifth outbreak, eight patients from the hematology ward at VGH were involved, testing positive over a period of 8 days. The accompanying epidemic curve is shown in Additional file 1: Fig. S2 (Supplement). While the affected patients had all been screened for SARS-CoV-2 on admission, they were not routinely re-tested until the first positive case occurred, despite often having—due to their underlying hematologic diseases—a long-term hospital stay. Four of the eight patients involved in the outbreak met the ECDC criteria for confirmed HA-COVID-19. According to medical staff, two of the patients later identified as SARS-CoV-2 positive had not been compliant with the hospital’s COVID-19 regulations (e.g. obligatory use of a face mask outside the patient room).

## Discussion

At VGH, we provided medical care without major restrictions in patient volume despite high COVID-19 case numbers in the population and limited single-room capacities. SARS-CoV-2 testing was used as part of a bundle of IPC measures. Our testing strategy of universal pre-admission screening and serial testing during hospitalization helped to identify patients with community-associated and healthcare-associated COVID-19. Compared to pre-admission screening, the diagnostic yield of short-interval re-testing was smaller.

The admission test positivity rate was 0.2% during the low and 2.3% during the high incidence phase. For both periods combined, 967 patients tested positive on admission. Without their timely identification through admission testing, these cases could have served as disease amplifiers in a hospital setting. Thus, universal pre-admission screening as part of our multi-tiered IPC strategy most likely contributed to the prevention outbreaks, especially given the challenges of a multi-patient room setting. However, in a low-incidence setting, the extra effort needed to conduct universal admission screening should be carefully weighed against its added value, especially when additional IPC measures are applied in parallel.

In the literature, variable admission test positivity rates have been reported. In line with our findings, the conferred benefit of universal pre-admission screening for the prevention of intra-hospital SARS-CoV-2 transmissions depends on the context, specifically the COVID-19 population prevalence. For example, Nakamura & Itoi’s results suggest that in a setting with low levels of community transmission, universal PCR testing prior to elective hospital admission might not be cost-effective due to its very low yield [31]. Krüger et al. found that universal admission PCR screening identified 27 out of 6940 patients as SARS-CoV-2 positive, which represents a positivity rate of 0.4% [32]. Overall, 67% of these patients could have been identified through clinical triage and targeted testing due to the presentation of symptoms or previous contact history with a COVID-19 case [32]. Consistent with these findings, Fassett et al. found the prevalence of SARS-CoV-2 infection among pregnant women universally screened on admission for delivery to be 0.4% [33]. However, all of them were asymptomatic at the time of diagnosis [33]. A much higher positivity rate was found by Sutton et al. in a setting with high community transmission rates at the early stages of the pandemic. Altogether, 29 (13.7%) out of 210 pregnant women admitted for delivery tested positive for SARS-CoV-2 [16]. They were all asymptomatic at the time of diagnosis. Sastry et al. found that universal pre-admission screening resulted in 79 (4.5%) out of 1755 patients testing positive for SARS-CoV-2 in a low incidence setting. Of these 79 patients, 12 (15%) were asymptomatic and 67 (85%) were symptomatic at the time of diagnosis [11]. Of note, these studies lack information regarding cut-off values used to define low and high incidence.

Regarding test conversions in our study population, only a small proportion of initially negative patients subsequently tested positive within 7 days after admission, 0.04% during the low and 0.5% during the high incidence phase, respectively. Early interval re-testing during the high incidence phase was able to find a total of 33 additional previously unknown cases with a newly acquired infection. However, the same strategy employed in a low incidence setting only yielded one additional previously unknown potential COVID-19 case. Thus, given the low yield and resource intensity of repeated testing shortly after admission in low incidence settings, other IPC strategies such as diagnostic testing of symptomatic individuals, testing of patients with known exposure to SARS-CoV-2 and the use of appropriate PPE should be prioritized. However, in times of PPE shortages and with sufficient testing capacity, serial testing could help create a sense of security among HCWs.

Consistent with our results, Adamson et al. found re-testing to be low-yield [17]. Among the 808 patients who initially tested negative, 11 (1.4%) subsequently tested positive. In their patient collective, reasons for re-testing were mostly symptom onset or worsening of already existing symptoms compatible with COVID-19 at the time of admission. Six out of the eleven positive patients were healthcare workers. The authors concluded that the decision for repeated testing should be guided by an ongoing risk for SARS-CoV-2 exposure (such as among healthcare workers) and the development of symptoms. There was no information on the background population prevalence provided. Another study that evaluated the diagnostic yield of early interval re-testing among initially negative patients with a high pre-test probability found that all of these 19 patients remained negative in the first 24 h [18], suggesting that results are unlikely to change within a day, even in a patient collective with ongoing clinical concerns. However, the study sample was very small.

In our study population, there was only one larger-scale outbreak that affected eight patients in total. The other four outbreaks involved only two patients that shared a room. This finding suggests that we were able to successfully identify and manage most COVID-19 cases before they could cause major outbreaks. In line with our findings of SARS-CoV-2 transmission occurring through shared patient rooms, Trannel et al. observed a secondary attack rate of 21.6% among patients sharing a room with a previously unidentified COVID-19 case. In their study, longer exposure was associated with a higher risk for transmission [4].

The one larger outbreak that occurred during the study period may have been caused by the non-adherence to the hospital’s infection prevention protocol because in this particular case, inpatients with a longer hospital stay had not been tested in weekly intervals. Of note, the IPC strategy at the time did not require visitors to show proof of a recent negative COVID-19 test result, and while visitors had to wear an N95 respirator for the duration of their visit, compliance with this regulation could not be monitored at all times. Therefore, it cannot be ruled out that a visitor was the source of the outbreak, infecting one of the inpatients who in turn infected other patients on the ward.

To minimize the risk of hospital-acquired COVID-19 among inpatients, serial testing of patients with a long-term hospital stay should be coupled with other measures such as vaccination of HCWs and access restrictions for visitors, only being granted access with a recent negative SARS-CoV-2 test result and/or proof of vaccination. In addition, patients and visitors alike should be repeatedly reminded to adhere to the hospital’s COVID-19 regulations (e.g. mandatory use of face masks while outside the patient room or during visitation).

The strength of our study is its large sample size. It also has several limitations. We could not differentiate between tests performed for screening purposes versus diagnostic testing due to symptoms. The joint evaluation of symptomatic and asymptomatic patients might have led to an overestimation of patients with a positive admission screening result. Conversely, patients who were sent home to self-isolate when testing positive shortly before their elective admission are not captured by our data analysis, which could also mean that the proportion of positively tested patients was actually higher. Further, inpatients might have occasionally been re-tested with antigen instead of PCR tests. Since this information was not available, we cannot rule out that potential antigen test results might have changed our outcomes.

With the available data, is not possible to say with certainty that serial testing can avert HA-COVID-19 and hospital outbreaks. Further, it is difficult to disentangle the impact of one single IPC measure—always applied as a bundle—on improved patient safety. However, our testing strategy possibly prevented exposure events because it identified previously unknown asymptomatic cases. It is worth remembering that our close-meshed serial testing strategy was developed in the context of maximum safety thinking, guided by the need to maintain essential health services in the midst of a pandemic. Future studies should evaluate the added benefit of different testing strategies compared to an IPC concept that does not include testing.

## Conclusion

Applied in conjunction with other IPC measures, our serial testing strategy was an effective tool for preventing nosocomial SARS-CoV-2 transmission and hospital outbreaks in a setting with a single patient-room capacity of 3% while running at full capacity, enabling the identification of previously unknown cases. Our findings show that it is possible to provide a safe environment for patient care, even when COVID-19 case numbers in the community are high.

## Supplementary Information

**Additional file 1.** Provision of safe patient care during the COVID-19 pandemic despite shared patient rooms in a tertiary hospital.

## Data Availability

The datasets used and/or analyzed during the current study are available from the corresponding author on reasonable request.

## Abbreviations

CA-COVID-19: Community-associated COVID-19
COVID-19: Coronavirus disease 2019
Ct: Cycle threshold
ECDC: European Centre for Disease Prevention and Control
HA-COVID-19: Healthcare-associated COVID-19
HCW: Healthcare worker
IPC: Infection prevention and control
IQR: Interquartile range
PPE: Personal protective equipment
RT-PCR: Reverse transcription polymerase chain reaction
SARS-CoV-2: Severe acute respiratory syndrome *coronavirus* type 2
VGH: Vienna General Hospital
