# Supplementary material for: Provision of safe patient care during the COVID-19 pandemic despite shared patient rooms in a tertiary hospital
Source: Antimicrob Resist Infect Control. 2022 Apr 21;11:61. doi: 10.1186/s13756-022-01091-1 (PMC9021561; doi:10.1186/s13756-022-01091-1)
Supplement: Supplementary file 1 — Additional file 1. Provision of safe patient care during the COVID-19 pandemic despite shared patient rooms in a tertiary hospital. [file 13756_2022_1091_MOESM1_ESM.docx]

Supplement

Figure S1. Laboratory-confirmed COVID-19 cases in Vienna


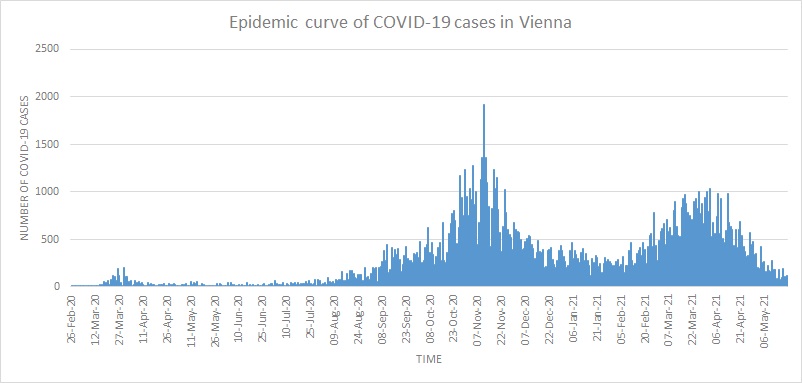


Figure S2. Epidemic curve of a suspected SARS-CoV-2 outbreak occurring on a ward at Vienna General Hospital


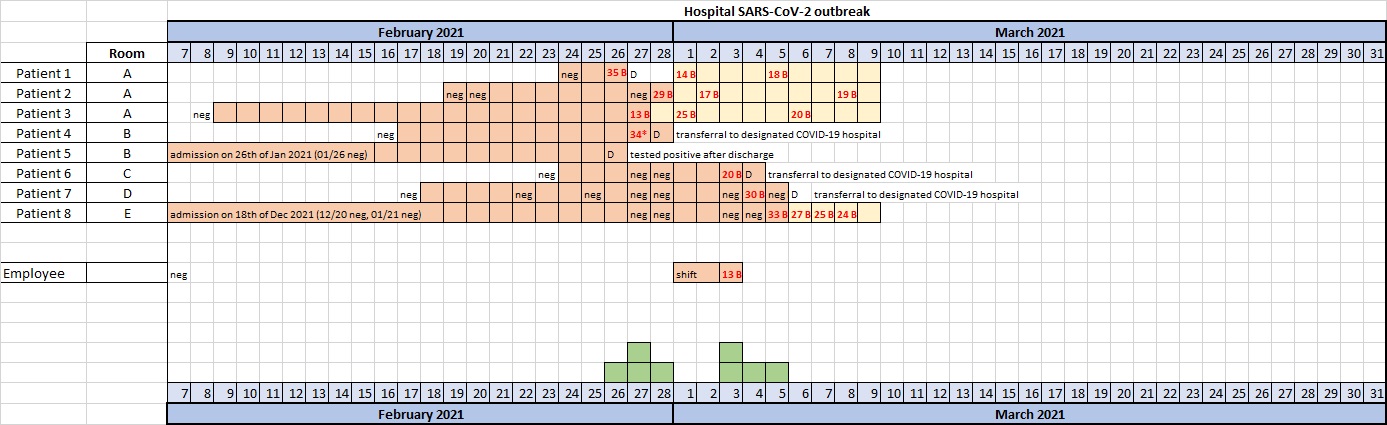


COVID-19 related outbreak at VGH on a ward with immunocompromised patients. The green boxes represent the absolute number of cases. The orange and yellow boxes represent patients over time. Each box reflects a day spent at the hospital. The orange color represents the ward where the outbreak occurred. The yellow color represents one of the hospital´s COVID-19 wards where some of these patients were transferred to upon testing positive. Red numbers indicate a positive test result, the numeric value represents the Ct-value; B=British variant (Alpha variant), D=discharge, neg=negative RT-PCR SARS-CoV-2 result, *=variant could not be analyzed.
